# Health Literacy Interventions to Improve Health Outcomes in Low- and Middle-Income Countries

**DOI:** 10.3928/24748307-20201118-01

**Published:** 2020-12-11

**Authors:** Salima Meherali, Neelam Saleem Punjani, Amynah Mevawala

## Abstract

**Background:**

Health care systems in many low- and middle-income countries (LMICs) face considerable challenges in providing high-quality, affordable, and universally accessible care. Feasible solutions to these issues require health literacy (HL) interventions for people who live in LMICs. Low HL is a significant problem in many LMICs because of the low levels of general literacy and poorly resourced and functioning health systems. A comprehensive understanding of HL interventions is essential to determine whether these interventions meet the health information needs of people who live in LMICs and to develop other effective HL interventions specifically for people who live in LMICs, improve health outcomes, and reduce inequalities.

**Methods:**

A medical research librarian developed and implemented search strategies to identify relevant evidence. Included studies needed to contain HL in LMICs component to understand or evaluate HL interventions that target people who live in LMICs. Two reviewers selected studies, conducted quality assessments, and extracted data by using standard forms. Discussion or third-party adjudication resolved disagreements. The collected data include the design of the study, type of HL intervention, target audience, theoretical influences, approaches to evaluating the intervention delivered, intervention received, intervention fidelity, intervention reach, data analysis, and study outcomes.

**Key Results:**

The reviewers systematically analyzed the data from 23 published research studies, including 20 quantitative, 1 qualitative, and 2 mixed-method studies, on HL interventions to improve the health outcomes in LMICs. The various HL interventions for different groups of the population depend on the health outcomes of the study. The reviewers identified four themes: traditional HL interventions, art-based HL interventions, interactive learning strategies, and technology-based HL interventions. The researchers of a few studies also used multicomponent interventions to improve the HL of the population.

**Discussion:**

Despite global improvements in health indicators over time, such as decreased mortality and morbidity, significant challenges remain regarding the quality of the delivery of health care in many LMICs. All of the HL interventions were effective and significantly improved the knowledge and awareness of the population. However, based on the literature review, the reviewers found significant evidence that only a limited number of HL interventions are delivered through innovative and technological learning strategies. In addition, the sustainability and scalability of these interventions is not clear. Therefore, future research on sustainability measures for effective HL interventions in LMICs is still needed. **[*HLRP: Health Literacy Research and Practice*. 2020;4(4):e250–e266.]**

The promotion of health literacy is critical to active and informed participation in health and health care (World Health Organization, 1998) and is a key action to reduce health inequalities. Health literacy (HL) is “the cognitive and social skills which determine the motivation and ability of individuals to gain access to, understand and use information in ways which promote and maintain good health” (World Health Organization, 1998). HL is the range of skills and competencies that people require to find, comprehend, evaluate, and use health information and concepts to make informed choices, reduce health risks, and improve quality of life (Adams et al., 2013). Improving people's access to health information and their capacity to use it effectively also empowers them to take a more assertive and more active role in their own, their family's, and their community's health care. In today's societies, HL is gaining increased attention, partly because of the recognition of the ethical imperative to engage patients in decision-making on their health and the growing evidence that patient participation has several benefits (Coulter, 2012; Elwyn et al., 2010; Vahdat et al., 2014; van de Bovenkamp et al., 2010). Examples of these benefits are increased patient knowledge, increased patient satisfaction with treatment decisions, reduced patient anxiety, and better treatment adherence (Stacey et al., 2011; Vahdat et al., 2014). Furthermore, care that is respectful of and responsive to patients' preferences, needs, and values is a key element of good quality (Institute of Medicine, 2001; World Health Organization, 1998).

Low HL is associated with inadequate knowledge about health and the health care system, poor access, and use of health services and increased hospitalization. This leads to poor health outcomes and health inequalities (Aboumatar et al., 2013; Al Sayah et al., 2013; Bostock & Steptoe, 2012). Previous studies have demonstrated correlations between low HL and increased hospital admissions and readmissions (Mitchell et al., 2012); poorer medication adherence and increased adverse medication events (Lindquist et al., 2012); less participation in prevention activities (von Wagner et al., 2007; World Health Organization, 1998); higher prevalence of health risk factors (Aung et al., 2012; Yamashita, & Kart, 2011); poorer self-management of chronic diseases and poorer disease outcomes (Schillinger et al., 2002); less effective communication with health care professionals (Schillinger et al., 2004); increased health care costs (Herndon et al., 2011); lower functional status (Wolf et al., 2005); and poorer overall health status (Howard et al., 2006; Tokuda et al., 2009), including increased mortality (Sudore et al., 2006). Studies have also suggested that the lack of HL significantly increases the burden of disease and reinforces health and economic inequalities (Berkman et al., 2011; Institute of Medicine, 2009; Institute of Medicine, 2011a; Institute of Medicine, 2011b; Poureslami et al., 2017).

Many low- and middle-income countries (LMICs) must deal concurrently with the challenges of combating communicable diseases and maternal and perinatal morbidity and mortality, as well as the rising burden of noncommunicable diseases (NCDs), including cardiovascular disease, cancer, diabetes, and mental and neurological disorders (Mayosi et al., 2012). In today's societies, HL is gaining more attention than ever before for many reasons. Contemporary health systems are multifaceted and can be challenging to navigate and understand. Education systems might not prepare people with the essential skills to relate successfully with modern health systems and information to improve their health (Parker, 2000; World Health Organization, 2013). Differences in HL contribute to health inequities and health outcomes (Dodson et al., 2015). Low HL leads to an unfavorable and unhealthy lifestyle and behavior; prevents the uptake of disease prevention and detection services; hinders self-management of chronic disease, compliance with medications, and understanding of provider communication; raises health care costs; and worsens existing inequities (World Health Organization, 2013). NCDs are a leading cause of deaths globally and particularly in LMICs and are associated with multiple modifiable behavioural risk factors (World Health Organization, 2011; World Health Organization, 2013). Low HL adversely affects the behavioral risk factors of NCDs, and vulnerable groups such as older adults, people with low levels of education, and racial and ethnic minority groups are also at a high risk for developing NCDs (World Health Organization, 2013).

Despite global improvements in health indicators over time, such as decreased mortality and morbidity, significant challenges remain about the quality of the delivery of health care in many LMICs. Health care systems in many LMICs face considerable challenges in providing high-quality, affordable, and universally accessible care. Feasible solutions to these issues require HL interventions for people who live in LMICs. Low HL is a significant problem in many LMICs because of the low levels of general literacy and poorly resourced and functioning health systems (Malik et al., 2017). Research on HL in LMICs revealed that more than 70% of adults who live in LMICs have inadequate HL, mainly because of fewer years of education and low financial status (Apolinario, 2014; Javadzade, 2012).

A comprehensive understanding of HL interventions is essential to determine whether these interventions meet the health information needs of people who live in LMICs and to provide accessible and equitable services to all. Furthermore, understanding the available HL interventions will help to develop other effective HL interventions specifically for people who live in LMICs, and improve health outcomes, and reduce inequalities. The goal of this study was to identify and synthesize the evidence on HL interventions to improve HL and health outcomes in LMICs.

## Methods

### Literature Search

A health research librarian developed a comprehensive search strategy that involved the content expertise of the research team to identify all relevant articles. The librarian searched the following electronic databases: MEDLINE, PubMed, Ovid MEDLINE, Cochrane Central Register of Controlled Trials, EPOC systematic review database, Cochrane Database of Systematic Reviews, Database of Abstracts of Reviews of Effects, Health Technology Assessment Database, HealthStar, Excerpta Medica, Cumulative Index of Nursing and Allied Health Literature, Psychological Abstracts, and Sociological Abstracts. To avoid publication bias, the librarian also reviewed dissertations and the reference lists of all articles for relevant citations and hand-searched key pediatric/child health journals and conference proceedings from each of the allied health professions included in the study according to the date (January 1985 to December 2018), language (English). The date restrictions reflect the emergence of evidence-based medicine/evidence-based practice and HL movements; the librarian purposively selected them to capture all relevant literature.

### Inclusion Criteria

The search included studies that met the following predetermined inclusion criteria: (1) primary research studies that included experimental, quasi-experimental, and nonexperimental designs (e.g., case study); (2) target populations that included all races, ethnicities, and cultural groups from all ages who lived in LMICs; (3) interventions/strategies with the primary purpose of improving or enhancing the HL of people or communities who lived in LMICs. Examples of potential interventions are written health information interventions (e.g., print material, brochures); educational workshops; technology-driven HL interventions (e.g., videos, audiotapes, mobile health); and targeted mass-media campaigns; (4) outcomes that included the use of health care and preventive services; receipt of recommended treatment; decreased emergency department visits and hospitalizations; increased knowledge/comprehension, self-efficacy, and health-related skills (e.g., ability to seek information and knowledge on how to take medications, self-monitoring, and how to access care); adherence to medications/ behavior; improved quality of life; decreased mortality and health care costs; and disparities by age, race, ethnicity, or culture.

### Study Selection

Two reviewers (N.S.P., A.M.) independently screened the search results to determine whether the study met the inclusion criteria. They rated each article as *include*, *exclude*, or *unclear* and retrieved the full texts of all articles classified as include or unclear for review. Two reviewers independently assessed the full reports of each potentially relevant study by using standard forms and predetermined inclusion criteria. If the two reviewers disagreed, a third reviewer (S.M.) discussed the discrepancies and made the final determination.

### Data Extraction

Two independent reviewers (N.S.P., A.M.) extracted relevant data from each study, including publication year, country, study design, sample characteristics, interventions, and outcomes (**Table 1**). They then checked the data for accuracy and completeness and discussed and resolved the discrepancies by referring to the original report and, if required, seeking third-party adjudication.

### Quality Criteria

To assess the methodological validity and quality of the studies, two reviewers independently assessed the quantitative and qualitative articles for retrieval for prior to inclusion in the review by using standardized critical appraisal instruments from the Joanna Briggs Institute (2017; Lee et al., 2010). We used multiple Briggs Institute checklists according to the types of studies, including the Checklist for Analytical Cross-Sectional Studies, Checklist for Case Control Studies, Checklist for Cohort Studies, Checklist for Quasi-Experimental Studies (nonrandomized experimental studies), Checklist for Randomized Controlled Trials (RCT), and Checklist for Qualitative Research. The reviewers resolved any disagreements through discussion and with a third reviewer (S. M.). The reviewers agreed that, for inclusion in the review, the studies must meet any of 7 of 10 methodological assessment criteria. We used The Mixed Methods Appraisal Tool checklist (Joanna Briggs Institute, 2017) for the quality appraisal of two mixed-method studies and included studies that met at least 75% of the quality criteria in the review.

### Data Analysis

We aggregated and analyzed the outcome data according to the type of HL intervention(s) and tabulated the HL strategies by using descriptive statistics. We also used descriptive (narrative) analysis to identify potential patterns (e.g., similarities, anomalies) in terms of targeted behaviors, study outcomes, and intervention effectiveness. This descriptive analysis satisfied two goals: (1) to examine successful strategies and (2) to explore what made different strategies effective, for whom, and under what circumstances (Pawson et al., 2005). We could not conduct meta-analyses because of the methodological and clinical heterogeneity of the studies.

## Results

**Figure 1.**
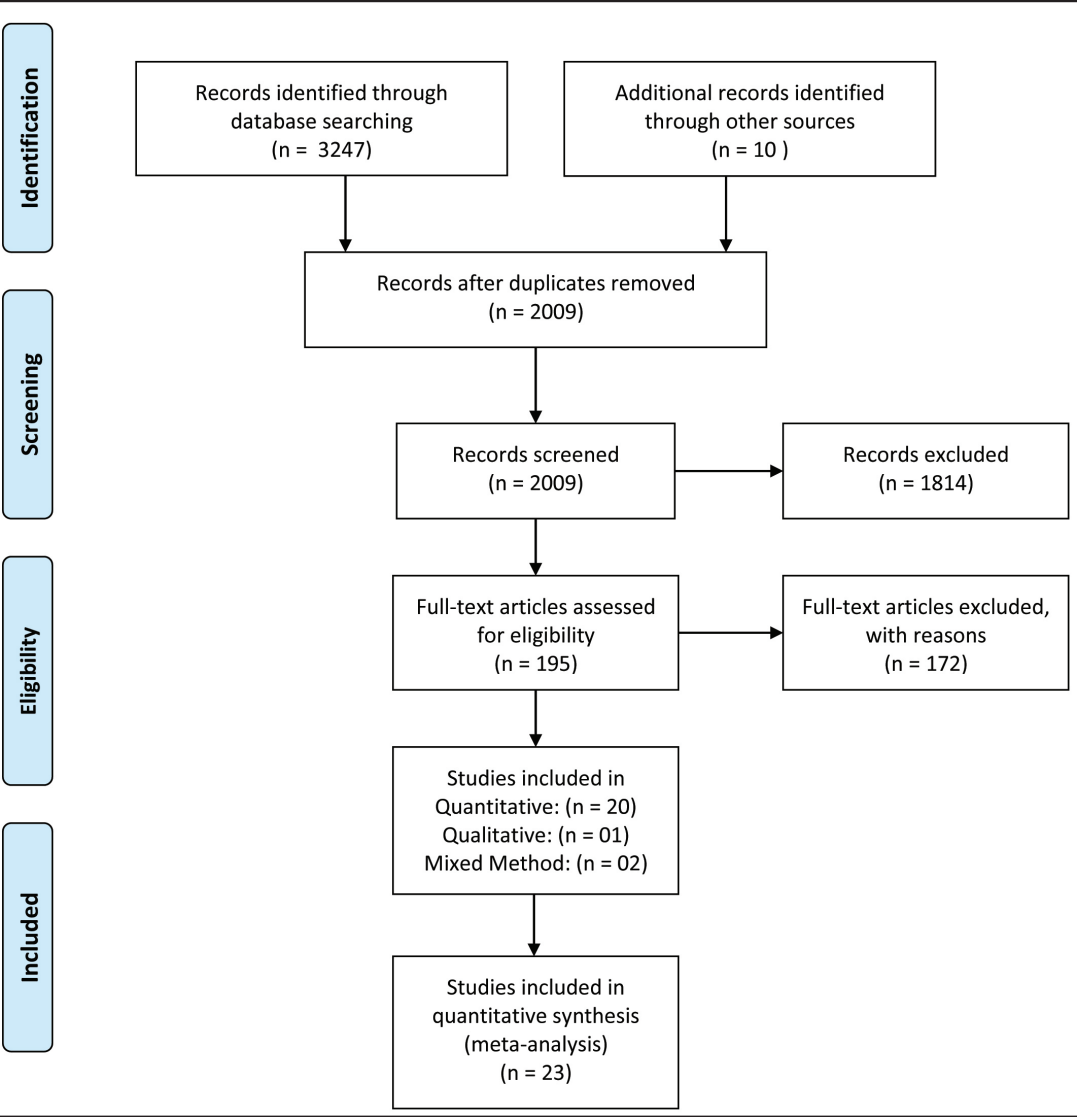
PRISMA (Preferred Reporting Items for Systematic Reviews and Meta-Analyses) diagram of the articles reviewed.

The initial search retrieved a total of 3,274 articles. After we removed the duplicates and articles written in languages other than English and reviewing abstracts with respect to the inclusion criteria, we considered a total of 2,009 studies relevant. After a full-text review and consultation among the authors, we included 23 articles in the final review and analysis using a PRISMA diagram (Moher et al., 2009) (**Figure 1**).We then summarized the findings from each article in table format and conducted a systematic analysis to extract major themes. The descriptive synthesis table that we formulated contains the textual descriptions of all of the findings. We grouped the extracted data and clustered them into categories to formulate themes and subthemes. Then we conducted a detailed analysis to evaluate the effectiveness of the HL interventions and their impact on health outcomes.

The 23 studies that we identified revealed HL interventions for various groups of people to improve the health outcomes in LMICs. **Table 1** summarizes the 23 selected articles according to the title, author, location of the study, purpose of the study, research design, sample, HL interventions, and outcomes. The researchers of many of the reviewed studies used quantitative design (*n* = 20); only one study involved a qualitative descriptive design (*n* = 1), and two involved mixed-method approaches (*n* = 2). The sample populations in the included studies were women, men, children, school and college students, adolescents, and people with disabilities.

Researchers conducted most of the studies in Asia and Africa, including Cambodia (*n* = 1) (Ngy et al., 2007), Egypt (*n* = 1) (Kharboush et al., 2011), Ghana (*n* = 2) (Lori et al., 2015; Lori et al., 2017), Guinea (*n* = 1) (McGinn et al., 2006), India (*n* = 5) (Braich et al., 2011; León et al., 2014; Mindlis et al., 2015; Noronha et al., 2013; Rajan & Nayak, 2014), Iran (*n* = 1) (Basir et al., 2017), Malaysia (*n* = 1) (Loh et al., 2009), Mongolia (*n* = 1) (Hikita et al., 2018), Pakistan (*n* = 2) (Ahmad et al., 2018; Ayub et al., 2015), Nigeria (*n* = 2) (Bella-Awusah et al., 2014; Hanass-Hancock et al., 2014), South Africa (*n* = 3) (Haricharan et al., 2017; Mhlongo et al., 2018; Hobday et al., 2015), Timor-Leste (*n* = 1) (Nabunya et al., 2015), Uganda (*n* = 1) (Khudanov et al., 2018), and Uzbekistan (*n* = 1) (Li et al., 2016). The expert librarian and lead author (S. M.), in consultation with the two co-authors (N. S. P. and A. M.), extracted the data.

We used various strategies and interventions to enhance the HL of different population groups, depending on the health outcomes of the study, and classified the interventions into four broad categories: traditional HL interventions, art-based HL interventions, active learning HL strategies, and technology-based HL interventions (**Table 2**).

Most of the 23 studies/articles involved traditional ways to deliver HL interventions (*n* = 10); however, the researchers of three studies used technology-based interventions (*n* = 3). The researchers used these innovative interventions recently, in 2016, 2017, and 2018. Six studies (*n* = 6) involved art-based interventions, and many researchers also used active learning strategies to enhance HL among different population groups (*n* = 4). Few researchers used multiple interventions to improve HL.

## Traditional HL Interventions

The available data indicate that most of the HL interventions had a limited focus on improving knowledge and gaining a cognitive understanding of health-related issues. Traditional interventions are strategies that are long established and have a longer history of implementation, particularly in LMICs. The main purpose of these interventions was to promote awareness regarding the curative and preventive measures of health-related issues. The traditional ways of delivering interventions were comprised of lectures, passive lessons, one-way delivery of information, distribution of pamphlets and leaflets, and health-education sessions with visual aids. In the traditional methods, researchers used both individual and group interventions to improve the HL of the population. In their research to improve the HL of mothers in Mongolia, Hikita et al. (2018) distributed maternal and child health handbooks to mothers whose literacy levels varied and investigated the use of handbooks among those mothers. The results indicate that this intervention was effective for literate women and that those who had learned to use the handbook were more likely to read it (adjusted odds ratio = 3.19, 95% confidence interval [1.68, 6.05]; adjusted odds ratio = 2.42, 95% confidence interval [1.31, 4.46], respectively). Similarly, to improve the HL of women on the risk factors and preventive practices regarding breast cancer, Kharboush et al. (2011) conducted 20 health-education sessions with women from underserved communities in Egypt. The outcomes indicate a significant increase in the mean knowledge score on breast cancer and the mean opinion score on some of the risk factors for breast cancer.

To improve the knowledge level of newly diagnosed women with breast cancer in Malaysia, Loh et al. (2009) delivered a self-management program in addition to the usual care to discuss medical, emotional, and lifestyle changes in a focus group with other women. At postintervention, the knowledge level of the experimental group of women who attended the self-management program had significantly improved. Moreover, to improve the HL of Iranian women with children age 12 to 36 months to prevent early dental caries in childhood, Basir et al. (2017) used educational interventions such as lectures and group discussions, along with standard well-baby care. The intervention improved HL knowledge, skills, and health behavior.

Braich et al. (2011) conducted an RCT in India in 2011 to improve the HL of cataract surgery patients. They divided the patients into two groups and educated the first group through verbal instruction only and the other group through verbal instruction and pictograms, which they gave the patients to take home. The results show that taking the pictogram home was the most effective way to educate patients who had low levels of literacy because it improved their overall HL and compliance with medication regimens. McGinn and Allen (2006) conducted another study with Sierra Leonean and Liberian women in refugee camps in Guinea to evaluate their HL on reproductive health knowledge. They used a traditional method to deliver an HL intervention through literacy classes that they taught for 2 hours twice a week for 6 months. The authors found that, postintervention, the women's reproductive health knowledge and literacy skills improved. The researchers of the above-mentioned studies concluded that verbal instructions, the use of pictograms, and literacy classes improve overall HL.

In India, Mindlis et al. (2015) evaluated social representations of depression in villages where an educational program had targeted mental illness and stigma and compared the results to those in control villages. The intervention group, who had a mental illness, received health education through workshops, counselling, treatment, and social and vocational rehabilitation. The intervention group reported improved levels of literacy on depression and decreased stigma, after the researchers controlled for other sociodemographic variables. Ngy et al. (2007) conducted a study in Cambodia, where antenatal women received HL interventions through an education program that included group-education sessions, individual consultation, and instructions to strengthen the functional knowledge and skills of the participants. This study shows that the HL sessions were positively associated with improved postpartum maternal health knowledge and fewer incidences of postpartum anemia and low birth weight.

Noronha et al. (2013) conducted an intervention study in Southern India to evaluate the effectiveness of a health information package in empowering pregnant women to adjust their health-care behaviors and take suitable actions to combat anemia in pregnancy. The pregnant women received the HL intervention through pamphlets, lectures, discussions, and question-answer sessions. This program significantly improved women's health-seeking behaviors and their perceptions of the significance of anemia during pregnancy. Rajan and Nayak (2014) also conducted a study in India to evaluate the effectiveness of self-instructional modules on mothers' knowledge of post-cesarean self-care after elective cesarean delivery who were admitted to hospitals in Mangalore. The researchers delivered the self-instructional module as an intervention and found that it effectively improved the knowledge of mothers post-cesarean delivery on postoperative self-care.

## Art-Based HL Interventions

Art-based interventions are strategies that involve nontraditional and innovative methods of delivering HL knowledge and awareness, such as drama classes, drawings, storytelling, and activities. Hanass-Hancock (2014) conducted a research study in KwaZulu-Natal, a rural community of South Africa, and examined the relationships among contextual factors such as caregivers, peers, and exposure to HL classes in relation to HIV knowledge, attitudes, and practice. She offered drama classes to adolescents as an HL intervention on HIV prevention and found that participation in the drama classes positively influenced sexual behavior and self-efficacy, as well as attitudes toward protective methods such as the use of condoms and delayed introduction to sexual activity.

Hobday et al. (2015) conducted a study in four primary schools in Aileu District in Timor-Leste; they trained local teachers and provided resources to students for lessons on eye health. The students, age 10 to 17 years, received an HL intervention through an activity book called “Healthy Eyes,” which contained children's drawings. Local artists modified the book, and it was translated into one of Timor-Leste's official languages, Tetun. The results show that the intervention was positively associated with improved eye-health knowledge, attitudes, and practices of the students.

León et al. (2014) conducted a study in Jharkhand, India, where they evaluated the effects of HL on women's decision-making power with regard to a family-planning intervention. They hired a nongovernmental organization specialized in street theater and puppet shows to deliver the HL intervention, which included awareness of and knowledge on contraceptive methods, communication among couples, family-planning decision-making, and women's reproductive rights. The HL intervention increased women's power to make decisions, and they became more empowered after they attended street theatre and puppet shows.

Ahmad et al. (2018) assessed the use of bilingual pictorial storybooks to improve school children's knowledge on preventing road-traffic incidents (RTIs) in Pakistan. The HL interventions included interactive discussions and a bilingual (Urdu and English) pictorial storybook on RTI prevention. The pictorial storybook improved the knowledge of primary school students in Pakistan on RTI prevention.

Mhlongo et al. (2018) assessed the impact of a health-education program in South Africa to improve knowledge on diabetes and reinforce preventive measures. They organized a science festival to educate school children; the health-education activities included presentations, posters, health models, word-search games, information leaflets, and a computer-based quiz. Post-intervention, the mean score on the school children' knowledge significantly increased.

Lori et al. (2015) conducted a study in Ghana and provided group antenatal care to improve women's HL by enhancing their capability to understand and implement health messages compared to that of women who received standard individual antenatal care. To improve their HL, they used demonstrations and role play strategies to emphasize the main messages and improve learning. They reinforced the HL messages by using pictorial “take-action cards.” The study showed positive outcomes in that the women who participated in group care improved their HL by gaining a better understanding of how to operationalize health-education messages.

## Interactive Learning Strategies

Interactive-learning HL interventions such as group discussions and peer-support programs, encourage learners to actively take the initiative and ownership to improve their health outcomes. Ayub et al. (2015) conducted a study in Pakistan and aimed to promote the civic responsibility and communication skills of college girls and enhance the HL on iron-deficiency anemia of students and community women. They conducted six small interactive sessions for 3 hours each. The HL interventions also included role play, pictorial pamphlets, posters, and a question-and-answer session. The pre- and post-assessments showed significant improvement in all three constructs of civic responsibility and in the participants' perceptions of their communication skills.

Lori et al. (2017) conducted a prospective cohort study in Ghana to determine whether group antenatal care would improve women's HL by enhancing their ability to comprehend and use health messages, compared to women who received routine, individual antenatal care. The interventions included storytelling, peer support, demonstration, and Teach-Back. Antenatal Ghanaian women who attended the group care demonstrated more HL in their improved understanding of health-education messages.

Bella-Awusah et al. (2014) evaluated the influence of a school-based mental health awareness program on secondary-school children age 10 to 18 years. This program was intended to enhance the literacy on mental health and decrease the negative opinions of people with mental illnesses in Nigeria. They delivered the mental-health literacy intervention in 3-hour sessions, followed by small- and large-group discussions. The results show that the small training workshops were productive and improved the mental-health awareness of young Nigerians.

Nabunya et al. (2015) measured the effects of a peer-mentorship program delivered with other supportive services on the HIV/AIDS knowledge, beliefs, and prevention attitudes of school-going, young people who were orphans in southern Uganda. They also conducted health workshops and the interactive peer-mentorship program to offer HL interventions to adolescents. As a result, the adolescent participants in the peer-mentorship program reported having more HIV/AIDS knowledge than the control group did.

## Technology-Based HL Interventions

Technology-based HL interventions are strategies to deliver health-related knowledge to target populations through technology (such as mobile devices, internet web-sites, digital devices). Haricharan et al. (2017) conducted a study in South Africa to determine whether a short message service (SMS)-based health promotion campaign would increase the knowledge of people who are hearing impaired on hypertension and healthy living. The authors also assessed the effectiveness and acceptability of SMSs. They found statistically significant improvements in knowledge and awareness on hypertension and healthy living among the deaf population.

In an RCT in Uzbekistan, Khudanov et al. (2018) assessed the effectiveness of an oral health-education program by means of a device that uses quantitative light-induced fluorescence technology to improve the oral hygiene and oral HL of adolescents. As an HL intervention, the researchers gave lessons on oral hygiene, demonstrated the device, and distributed leaflets. The study showed great improvements in the interventional group compared to the control group regarding the plaque index (95% confidence interval [0.46, 0.07]; *p* < .05), knowledge on oral health (95% confidence interval [19.4, 28.8]; *p* < .05), attitude (95% confidence interval [16.7, 20.2]; *p* < .05), and behavior of adolescents (95% confidence interval [19.9, 30.5]; *p* < .05).

In a study of Chinese expatriates in Niger, Li et al. (2016) evaluated the participants' HL on malaria to create a health-education program for the prevention and treatment of malaria among vulnerable travelers and expatriate employees; they used social media platform accounts and assessed users' satisfaction regarding their HL on malaria. To improve the HL intervention, the researchers used social media to deliver free instant messages on malaria. The knowledge, attitudes, practice, skills, and overall HL of the experimental population increased significantly compared to those of the controls; the statistically significant difference was *p* < .01.

## Discussion

HL is a growing area of public health research in LMICs in addition to the Western context to improve health outcomes and quality of life. The evidence that researchers have collected provides insights into the gaps in research in the context of LMICs, and they have made recommendations for future research. To our knowledge, this is the first systematic review of research on the evidence on HL interventions in the context of LMICs.

In this systematic review we have synthesized the evidence on strategies and interventions and their effectiveness in improving the HL of varied populations in LMICs. The researchers delivered four different classifications of HL interventions: traditional HL interventions, art-based HL interventions, active learning strategies, and technology-based HL interventions. Moreover, they targeted different populations such as children, expectant mothers, adolescents, students, patients with chronic diseases, and people with disabilities to assess the HL interventions. The focus of the interventions was to promote and maintain good health and prevent disease and complications to achieve a better quality of life. The topics of the HL ranged from sexual and reproductive health to antenatal and postnatal care, oral health, breast cancer, malaria, RTIs, chronic diseases such as hypertension and diabetes, cataracts, mental health, and communicable diseases such as HIV. However, the researchers aimed most of the HL interventions at improving women's sexual and reproductive health and well-being.

Overall, the most used methods of delivering health-related awareness and knowledge are traditional and include lectures, passive lessons, one-way delivery of information, distribution of pamphlets and leaflets, and health-education sessions using visual aids. Sufficient evidence has shown that the HL strategies of lectures and discussions have the potential not only to improve health-related knowledge and outcomes (Brainard et al., 2016; Brijnath et al., 2016; DeWalt & Hink, 2009; Jacobs et al., 2016; Manafo & Wong, 2012; Perazzo et al., 2017; Pignone et al., 2005), but also to improve comprehension (Sheridan et al., 2011) and patients' adherence to treatment (Miller, 2016; Perazzo et al., 2017). In addition, written and printed health-education materials are easy to read and understand and improve the health awareness of children and adults (DeWalt & Hink, 2009; Pignone et al., 2005). However, a challenge of the use of traditional methods is low literacy levels, which is a significant problem in LMICs that can lead to poorer health outcomes (Budhathoki et al., 2017; Jacobs et al., 2016; McCray 2005; van der Heide et al., 2014). The researchers of the studies included in this review reported a positive impact of traditional HL interventions on health outcomes. However, it is not clear in the studies whether these interventions are effective for people with low literacy levels.

This review suggests that many researchers have used active learning strategies to deliver HL knowledge. These interventions do not merely improve awareness and information, but also empower participants to take charge of their own health and well-being. These findings are consistent with substantive evidence from Brega et al. (2015) and Chin et al. (2012) that group- or peer-education programs deliver the most knowledge. Moreover, these types of interactive programs help to access and use health information (Manafo & Wong, 2012).

HL interventions that involve the internet or eHealth technologies have undoubtedly improved the community's access to health care information in recent years (Hur et al., 2015; Mackert et al., 2016). The recipients of this information are patients as well as the healthy population (McCray, 2005). This review has shown that eHealth technologies are the least-used methods of delivering health education in LMICs. Only three studies in the review incorporated strategies such as social media, SMS, and the device that uses quantitative light-induced fluorescence technology (Khudanov et al., 2018). This approach helps learners to participate actively and facilitates the innovation of instructional methods. Technology-based strategies have the potential to tailor learning to the needs of people (L'Engle et al., 2016; Pantoja et al., 2017).

The use of innovative and creative health interventions improve the HL outcomes of the population. Although a few researchers used art-based interventions to deliver HL education, they integrated and combined many other interesting and innovative ways to deliver HL, including drama classes, puppet shows, drawing books, exhibitions, computer games and quizzes, street theater, and storybooks. Strong evidence supports the use of strategies such as street theater, puppet shows, age-appropriate colorful drawing books, and storytelling to improve the HL of and empower participants (Ahmad et al., 2018; High et al., 2000; León et al., 2014). Previous research has also illustrated the power of art- and narrative-based forms to communicate with, engage, and influence people (Hartling et al., 2010). However, limited research from LMICs has involved the use of art and narrative forms to transfer research-based health knowledge to patients and their families.

The researchers of a few of the studies included in the review used multiple strategies/interventions to promote HL. Evidence suggests that multifaceted interventions have the potential to affect a larger population with limited resources. The researchers of three articles used multifaceted interventions—those that include more than one strategy—to provide HL education. The researchers of these studies combined multicomponent approaches to enhance their participants' engagement and improve health outcomes. This review has shown that multifaceted interventions are more likely to improve health outcomes. These findings correlate with the study outcomes of Sheridan et al. (2011), who suggested that HL interventions that combine several methods to improve health awareness, such as written and visual resources that are easy to understand, video tutorials, and HL training, enhance patients' understanding and appropriate use of health care (Scott et al., 2012). However, these findings contradict those of Squires et al. (2014), who found that multifaceted interventions are not more effective than single interventions.

Overall, the literature review reveals that HL interventions improve the health outcomes of target populations. In addition to improving knowledge from health information, they also contribute to major lifestyle changes. Thus, improving HL might have positive health outcomes at both the individual and the community level because it will enhance health and well-being and decrease unnecessary health care expenditures. Moreover, tailoring interventions to the needs of populations such as those with low literacy or limited exposure to the use of technologies promotes patient engagement (Jacobs et al., 2016). Teaching participants how to access appropriate health information will enable them to seek health information more effectively (Car et al., 2011).

## Recommendations

We found substantial gaps in research evidence of the effectiveness of HL interventions and their outcomes in LMICs. Using successful and effective HL interventions and strategies will improve health outcomes and help to achieve sustainable developmental goals, including the goal to “ensure healthy lives and promote well-being for all at all ages” (Barredo et al., 2015). Based on the findings of this systematic review, we recommend the development of innovative and interactive interventions to improve HL and the development of guidelines for implementation.

In addition, this review has shown the need to improve HL on communicable, noncommunicable, and preventable diseases and for relevant health care professionals and stakeholders to develop interventions. Stakeholders from both the public and the private sector must be involved in developing strategies at the national, regional, and global level. We highly recommend the use of available resources and technologies to incorporate innovative strategies to improve HL skills and knowledge. Moreover, globally, we recommend that lessons learned, best practices, information, and computing technology be used as tools to improve HL in the region. Significantly, with respect to the various social, economic, and political contexts of different countries and their respective health issues, we recommend that the capacity of health care professionals to develop HL interventions be improved by involving them actively.

## Study Limitations

It is difficult to generalize the findings of this systematic review because only 23 published studies met the selection criteria. The approach that we used might have resulted in the omission of studies if the authors did not use the term “health literacy” in their work. Moreover, we were able to capture data only from major medical sources. Research on implementation programs and policy initiatives is often not available in the medical literature. Nonetheless, this is the first review to have explored the impact of different interventions and communication strategies on the HL of people who live in LMICs.

## Conclusion

Much remains to be done to improve HL in LMICs in this era of sustainable development goals. Many effective interventions that involve multiple strategies are required to deliver health-related information. Evidence from this systematic review shows the effectiveness of a range of approaches to HL delivery. The lessons learned from this systematic review suggest the need to improve HL interventions by using innovative approaches. Moreover, involving health service providers and community in co-designing HL interventions and materials is important to improve HL outcomes. The current review presents evidence from an array of studies that provide exemplars and hopefully the impetus to implement HL interventions at the population scale needed to improve health outcomes in LMICs. Such an effort will require considerable work such as developing local content, coordinating governments and private organizations, providing funding to sustain the impact of HL projects, and empowering community members to take the initiative to improve their health.

## Figures and Tables

**Table 1:** Health Literacy Interventions in Low- and Middle-Income Countries

**Study**	**Design**	**Setting or Region**	**Purpose/Area of Study**	**Health Literacy Intervention**	**Target Population**	**Outcome**
Ahmad et al. (2018)	Quantitative (pre-test and post-test)	Karachi, Pakistan	The study assessed the use of bilingual pictorial (Urdu and English) storybooks to improve road-traffic incidents (RTIs) prevention knowledge among school children	Interactive discussion	Children in grades 4 and 5	Discussions using bilingual pictorial storybooks helped primary school children in Pakistan grasp knowledge of RTIs prevention
Ayub et al. (2015)	Interventional exploratory study using a mixed methods approach	Public Girls' Degree College (Pakistan)	The objectives demonstrated the effectiveness of service-learning in fostering civic responsibility and communication skills in college students and to increase health literacy regarding iron deficiency anemia (IDA) among students and women in the community	Small interactive group sessions	College students	Students showed significant improvement in all three constructs of civic responsibility and in perceptions of their communication skills
Basir et al. (2017)	Quantitative, experimental, and control group study	Ahvaz, Iran	The study evaluated an intervention for preventing early childhood caries (ECC) in a pediatric population	Well baby care, educational interventions, and lecture and group discussion	Women with children age 12 to 36 months without dental caries	Interventions had positive effects on the perceived threat, health literacy, and health behaviors; and the intervention could reduce the incidence of ECC
Bella-Awusah et al. (2014)	Quantitative pre-intervention, immediate post-intervention, and 6-month post-intervention questionnaire	Ibadan Southwest, Nigeria	The study aimed to assess the effect of a school-based mental health awareness program on increasing mental health literacy and reducing negative views about people with mental illness	A 3-hour mental health awareness session	Secondary school students	Brief training workshops potentially produced small but positive changes in the mental health knowledge of young Nigerians
Braich et al. (2011	Quantitative and randomized control trial (RCT)	India	The study examined the effectiveness of pictograms in educating patients with low health literacy to improve adherence to postoperative cataract regimens	Three groups educated differently regarding medication use and frequency of dose	Patients from across India	Patients taking pictograms home proved to be the most effective way to educate them, and it increased adherence to regimens by 28 days or more
Hanass-Hancock (2014)	Quantitative survey	Rural community in South Africa	The study investigated the association between contextual factors, such as caregivers, peers, and exposure to the literacy classes regarding HIV knowledge, attitudes, and practice in school-age children	Literacy and drama classes	Male and female adolescents	Contextual factors may influence sexual behavior and self-efficacy as well as attitudes toward condom use
Haricharan et al. (2017)	Quantitative questionnaire	South Africa	The study aimed to assess whether a short message service (SMS)-based health promotion campaign could improve people who are hearing impaired knowledge of hypertension and healthy living	SMS-based information campaign	People who are hearing impaired	Statistically significant improvement in overall knowledge about hypertension and healthy living was attained
Hikita et al. (2018)	Population-based cross-sectional study	Bulgan Province, Mongolia	The study investigated the use of a Maternal and Child Health (MCH) handbook, and related factors	MCH handbook	Women with children born between January and December 2010	Mothers with middle or high educational attainment were more likely to have read the handbook than those with low educational attainment
Hobday et al. (2015)	A descriptive, mixed-methods design, questionnaires, and interviews	Four primary schools in Aileu District, Timor-Leste	The study assessed whether there was an improvement in the knowledge, attitudes, and practices of students after the Healthy Eyes in Schools Project intervention	Training and resources to implement nine lessons about eye health	Students in grades 5 and 6	Students attained an improvement in eye health knowledge
Kharboush et al. (2011)	A pre-/post-test interventional study	Egypt	The study aimed to determine the effectiveness of a health education program on raising the knowledge related to breast cancer (BC), its risk factors, and some related preventive practices among women	Health education sessions	Women age 30–65 years	Participants increased their mean knowledge score regarding BC and the mean opinion score regarding some BC risk factors
Khudanov et al. (2018)	Quantitative RCT	Uzbekistan	The study aimed to determine whether an oral health education program using an imaging device based on quantitative light-induced fluorescence (QLF) technology could improve the oral hygiene status and oral health literacy of adolescents	Education and training on dental plaque removal using the QLF device	Adolescents age 14–16 years	Statistically significant improvements in the experimental group compared to the control group in the plaque index was attained
León et al. (2014)	Quasi-experiment	India	The study aimed to determine whether literacy moderated the effects on women's decision-making power of the family planning intervention and whether literacy and women's decision-making power together moderated the effects of the intervention on met need for contraception	Family planning intervention, education via community theater performances	Married women ages 15–49 years	Women's normative beliefs concerning wives' power in decisions regarding money earned and visits to relatives and friends vis-à-vis their husbands' power were increased
Li et al. (2016)	Randomized, unblinded, controlled trial	Niger	The study aimed to investigate the malaria health literacy level of Chinese expatriates in Niger and to develop a health education program for the prevention and treatment of malaria among travelers who are not immune	Malaria health literacy	Chinese expatriates in Niger	Participants exhibited a greater change in knowledge, attitude, practice, skills, and overall health literacy
Loh et al. (2009)	Quantitative	Malaysia	This article described the knowledge of Malaysian women regarding BC and how participation in a self-management program can improve the situation	Self-management program	Women newly diagnosed with BC	Post-intervention showed significantly better knowledge within the experimental group
Lori et al. (2017)	Prospective cohort	District hospital in Kumasi, Ghana	The study examined whether exposure to group antenatal care increased women's health literacy by improving their ability to interpret and use health messages compared to women who received standard, individual antenatal care	Group antenatal care	Pregnant women	Women participating demonstrated improved health literacy by exhibiting a greater understanding of how to operationalize health education messages
Lori et al. (2015)	Prospective cohort	Ghana	The study examined the usefulness and feasibility of providing focused antenatal care in a group setting to improve patient-provider communication, patient engagement, and health literacy	Antenatal care modules	Pregnant women	Significant difference between women enrolled in group antenatal care versus individual antenatal care for preventing problems before delivery, understanding when to access care, birth preparedness and complication readiness, intent to use a modern method of family planning postpartum, greater understanding of the components of breast-feeding and lactational amenorrhea for birth spacing, and intent for postpartum follow-up was apparent
McGinn & Allen (2006)	Qualitative	Guinea	The study researched the Reproductive Health Literacy Project among Sierra Leonean and Liberian women in refugee camps	Literacy classes	Sierra Leonean and Liberian women	Participants had a high level of reproductive health knowledge after participation, and reported an increase in literacy skills
Mhlongo et al. (2018)	Quantitative (pre-intervention) and post-intervention	South African National Science Festival	The study determined the effects of a health education program on increasing knowledge about diabetes and encouraging preventive measures	A public health education exhibition	School children	Participants experienced a significant difference in their mean scores after the intervention
Mindlis et al. (2015)	Cross-sectional study	Rural Gujarat, India	The study compared social representations of depression in villages where educational programs have targeted mental illness and stigma versus control villages	Educational interventions	Male and female participants age 18–78 years	Higher levels of literacy regarding depression and lower levels of stigma, after adjusting for all other sociodemographic variables, were demonstrated
Nabunya et al. (2015)	Quantitative	Southern Uganda	This study evaluated the effect of a peer mentorship program provided in addition to other supportive services on HIV/AIDS knowledge, beliefs, and prevention attitudes	Peer mentorship program	School-going adolescents who were orphaned	Results indicate that when controlling for socioeconomic characteristics, adolescents who participated were more likely than nonparticipants to report increased scores on HIV/AIDS knowledge
Ngy et al. (2007)	Survey	Phnom Penh, Cambodia	The study aimed to examine the use of antenatal care with comprehensive health education qualified for the health of mothers and infants during perinatal and postpartum periods	Antenatal care with comprehensive health education	Pregnant women	Results show the solid utilities of qualified antenatal care for perinatal health
Noronha et al. (2013)	Quasi-experimental (pre-test and post-test)	Southern India	The study determined the effectiveness of a health information package in terms of empowering the pregnant women to modify their health care behavior and take appropriate action to combat anemia in pregnancy	Validated planned educational program with visual aids and iron supplementation	Pregnant women with anemia	Health education contributed significantly in modifying health-seeking behavior and perception about significance of anemia
Rajan & Nayak (2014)	One group pre-test post-test design which was a pre-experiment	Mangalore, Indian state of Karnataka and South India	The study determined the effectiveness of a self-instructional module on knowledge of postoperative self-care for mothers undergoing elective cesarean deliveries	Self-instructional module	Mothers who underwent elective cesarean delivery	Participants knowledge of post-operative self-care was improved

**Table 2:** Major Interventions and Strategies Generated to Enhance Health Literacy

**Study**	**Traditional**	**Art-Based**	**Interactive**	**Technology Based**
Ahmad et al. (2018)		✓		
Ayub et al. (2015)		✓	✓	
Basir et al. (2017)	✓		✓	
Bella-Awusah et al. (2014)		✓	✓	
Braich et al. (2011)	✓			
Hanass-Hancock (2014)		✓		
Haricharan et al. (2017)				✓
Hikita et al. (2018)	✓			
Hobday et al. (2015)		✓		
Kharboush et al. (2011)	✓			
Khudanov et al. (2018)				✓
León et al. (2014)		✓		
Li et al. (2016)				✓
Loh et al. (2009)	✓			
Lori et al. (2015)		✓		
Lori et al. (2017)		✓	✓	
McGinn & Allen (2006)	✓			
Mhlongo et al. (2018)		✓	✓	
Mindlis et al. (2015)	✓			
Nabunya et al. (2015)			✓	
Ngy et al. (2007)	✓			
Noronha et al. (2013)	✓			
Rajan & Nayak (2014)	✓			
